# Lab-on-a-chip device for microfluidic trapping and TIRF imaging of single cells

**DOI:** 10.1007/s10544-025-00739-0

**Published:** 2025-03-14

**Authors:** Dustin Dzikonski, Riccardo Zamboni, Aniket Bandyopadhyay, Deepthi Paul, Roland Wedlich-Söldner, Cornelia Denz, Jörg Imbrock

**Affiliations:** 1 Institute of Applied Physics, University of Münster, Corrensstr. 2, 48149 Münster, Germany; 2 Institute of Cell Dynamics and Imaging, University of Münster, Von Esmarch Str. 56, 48149 Münster, Germany; 3 Physikalisch-Technische Bundesanstalt, Bundesallee 100, 38116 Braunschweig, Germany

**Keywords:** Single cell trapping, TIRF imaging, Two-photon polymerization, Microfluidics

## Abstract

**Abstract:**

Total internal reflection fluorescence (TIRF) microscopy is a powerful imaging technique that visualizes the outer surface of specimens in close proximity to a substrate, yielding crucial insights in cell membrane compositions. TIRF plays a key role in single-cell studies but typically requires chemical fixation to ensure direct contact between the cell membrane and substrate, which can compromise cell viability and promote clustering. In this study, we present a microfluidic device with structures designed to trap single yeast cells and fix them in direct contact with the substrate surface to enable TIRF measurements on the cell membrane. The traps are fabricated using two-photon polymerization, allowing high-resolution printing of intricate structures that encapsulate cells in all three dimensions while maintaining exposure to the flow within the device. Our adaptable trap design allows us to reduce residual movement of trapped cells to a minimum while maintaining high trapping efficiencies. We identify the optimal structure configuration to trap single yeast cells and demonstrate that trapping efficiency can be tuned by modifying cell concentration and injection methods. Additionally, by replicating the cell trap design with soft hydrogel materials, we demonstrate the potential of our approach for further single-cell studies. The authors have no relevant financial or non-financial interests to disclose and no competing interests to declare.

**Graphical abstract:**

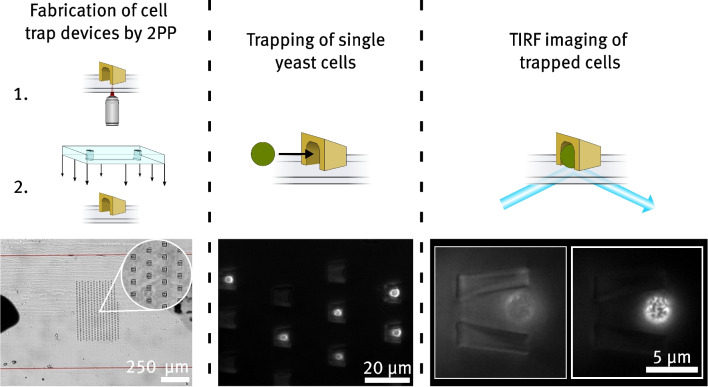

**Supplementary Information:**

The online version contains supplementary material available at 10.1007/s10544-025-00739-0.

## Introduction

With the technological advancement of various imaging techniques throughout the last decades, single cell analysis has continuously gained importance for the investigation of biological phenomena, as it offers unprecedented insight into cellular functions, interactions, and heterogeneities (Ding et al. 2020; Labib and Kelley 2020; Mattiazzi Usaj et al. 2021; Sun et al. 2023). While conventional methods for assessing cells typically quantify only the average biophysical properties of an entire cell culture, single-cell imaging uncovers the heterogeneity and distinct characteristics within individual cells of the sample (Hu et al. 2016). Staining individual biomolecules of cells, such as DNA or specific proteins, enables the quantification of their composition in both temporal and spatial contexts, providing insights into cell development, the function of cellular compartments, and the molecular mechanisms underlying pathogenesis. To visualize the comparatively weak signal from such small features, researchers developed various elaborate methods to suppress background signals from, e.g. autofluorescence of the cell and to surpass the diffraction-limited resolution of conventional fluorescence microscopy (Yang et al. 2021). One approach is utilizing Total Internal Reflection Fluorescence (TIRF) microscopy, in which the sample is illuminated with a laser beam under an angle larger than the critical angle (Fish 2022; Funatsu et al. 1995). As a result, the excitation light is fully reflected by the sample, and only the evanescent waves at the surface reach the sample to excite fluorescent molecules. Thus, excitation is usually limited to a depth of a few nanometers in the specimen, making it an excellent technique to visualize cell membranes or cell walls, while signals from other, deeper regions within the cells are suppressed (Alavizargar et al. 2022). Therefore, obviously, one requirement for successful TIRF imaging on single cells is to ensure that they are in direct contact with the substrate. For cells that do not adhere to the substrate intrinsically, fixing the cells prior to imaging is required. This can be accomplished through biochemical reactions such as using paraformaldehyde to covalently bond free amino groups of membrane proteins to functional groups on the substrate surface or by dehydration with organic solvents (Irgen-Gioro et al. 2022; Rodig 2020). However, none of these methods is entirely non-invasive, often introducing structural and biochemical alterations, cell toxicity, or additional sources of autofluorescence (Bhat and Hussein 2021). Additionally, these methods require extra processing steps that can be costly, time-consuming, and reduce experimental reproducibility. Furthermore, in these approaches, cells adhere to random positions on the substrate, which may lead to cell clustering and complicate automated analysis, as immobilized cells must first be located.

**Fig. 1:**
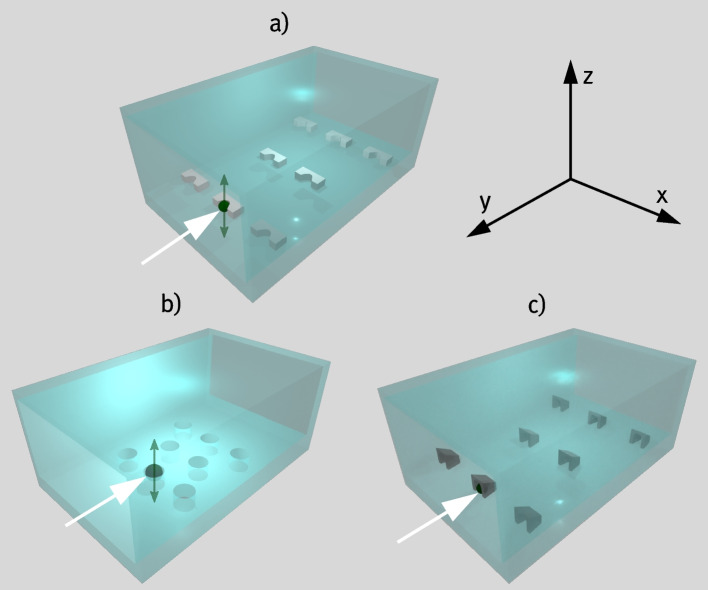
Visualization of different strategies to trap cells in microfluidic channels. Green spheres indicate cells, gray elements the trap structures and white arrows the flow direction, respectively. a) shows simple pseudo 3D-structures that prevent cells to pass. b) shows microwells at the bottom of the channel. c) illustrates the strategy applied in this work in which fully three-dimensional cell trap structures fix trapped cells in all directions

To avoid these disadvantages, our approach uses microfluidic trapping to mechanically fix cells at the substrate surface, making them well-suited for TIRF microscopy. Among the numerous techniques used to trap single cells, microfluidic trapping is one of the most widespread approaches (Deng et al. 2019; Narayanamurthy et al. 2017). This is because it typically relies on mechanical interactions of the cell with the flow of the medium and with obstacles made of biocompatible materials, such as polydimethylsiloxane (PDMS) or hydrogels (Barwig et al. 2024; Hu et al. 2019; Narayanamurthy et al. 2017). In contrast to other methods such as optical, magnetic, or chemical cell trapping, no additional physical or chemical signals that might affect the cells are required. Furthermore, passive hydrodynamic cell trapping enables single cell analysis with high throughput, usually without requiring any specialized equipment, making it applicable to many practical cell biology studies. In this context, the most common approach is using a microfluidic device made by replica molding containing pillars or pocket-like structures acting as a defined obstacle for cells which are flowing through the device (Banaeiyan et al. 2013; Carlo et al. 2006; Deng et al. 2019; Hu et al. 2019; Ryley and Pereira-Smith 2006). As fabrication techniques of soft lithography are constantly developed further, trapping devices can exhibit further functionalities such as separating budding cells from the daughter cells (Crane et al. 2014), bringing two types of cells in direct vicinity to each other (Dura et al. 2014; Skelley et al. 2009), sorting cells by size (Zhu et al. 2020) or trapping and releasing cells based on environmental cues (Barwig et al. 2024). Despite the vast potential of standard microfluidic devices to separate and trap single cells, they usually fail to fix the cells entirely without any residual motion. A common problem of replica molding fabrication is that it cannot easily provide any structural complexity of included elements in the direction perpendicular to the substrate surface (from here on referred to as the *z-direction*). Hence, the trapped cells are never fully fixed in all spatial directions, but the motion is only inhibited laterally. Cell trap designs that block the way of cells perpendicular to the z-direction, such as V-shaped pockets (as shown exemplary in Fig. 1a)), require constant flow of the surrounding medium for the cells to remain in the trap. As a result, shear stress can cause unwanted cell motion, especially in the z-direction, where cell motion is not prevented. In devices that utilize vertical cell traps, such as microwell arrays (as shown exemplary in Fig. 1b)), choosing the correct dimensions can pose a difficult challenge (Wang et al. 2024). This is because cells might escape pockets that are too small as they are still exposed to horizontal flow, which lowers the efficiency of the trapping device. However, large pockets can again lead to cell motion within, making the device unsuitable for investigations requiring long integration times, such as TIRF microscopy. With the trapping efficiency being subject to the respective cell size, this approach has limited applicability for single cell analysis.

In this work, we combine the advantages of horizontal and vertical trapping and additionally create a defined obstacle for trapped cells in the z-direction, reducing any residual motion to a minimum. The concept is illustrated in Fig. 1c). We fabricate fully three-dimensional cell traps using two-photon polymerization (2PP). This additive manufacturing technique is based on the simultaneous absorption of two photons from a femtosecond laser triggering the photopolymerization of a monomer to create structures with small feature sizes (Harinarayana and Shin 2021; Jaiswal et al. 2023). As the underlying process of two-photon absorption is spatially highly confined in a small region around the laser’s focus spot, 2PP enables the fabrication of highly complex features in all spatial directions with submicrometer resolution. Notably, 2PP allows the creation of arched structures on the micrometer scale, which is not easily achieved by other methods (Kawata et al. 2001). Additionally, 2PP supports processing a wide range of biocompatible materials such as hydrogels, making it an invaluable tool for numerous bioengineering applications (Song et al. 2019). Here, we use 2PP to fabricate free-standing three-dimensional cell traps on standard coverslips with sizes tailored to trap yeast cells. For this, we use the biocompatible, commercially available photoresist *Ormocomp*^®^. The cell traps are embedded in a microchannel by bonding PDMS molds onto the coverslip afterward. We optimize the overall shape and specific dimensions of the cell traps by injecting yeast cells into the device and analyzing the cell motion inside the respective traps as well as the efficiency of specifically trapping single cells. The trapping efficiency of a device with an optimized trap design is investigated with different cell concentrations. Furthermore, trapping efficiency is investigated for the case that the cells are injected once in the device with a pipette, and for the case that they are exposed to constant flow of the cell solution. As a proof-of-principle, we use yeast cells whose membrane proteins are stained with green fluorescent protein (GFP) to perform TIRF microscopy with trapped cells. Yeast cells, with their relatively round shape and rigid structure due to the presence of a thick cell wall (Feldmann 2012), are typically challenging to image using TIRF microscopy without permanent fixation to the substrate. This makes them an excellent model organism for testing the effectiveness of the fabricated traps in securing cells in direct contact with the glass substrate. Finally, we demonstrate the versatility of the presented approach by fabricating a similar device with cell traps made of hydrogel and demonstrate its potential for TIRF imaging.

## Materials and methods

**Fig. 2:**
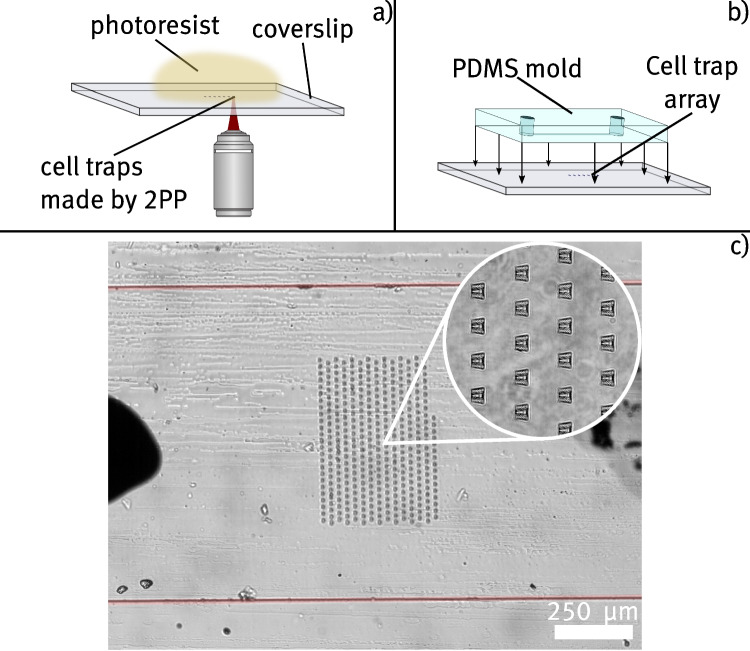
Schematic representation of the process used to produce microchannels containing structures made with 2PP. a) depicts the 2PP process, which is performed by scanning the focus of a pulsed near infrared laser across a drop of photoresist cast on a standard coverslip. b) shows the bonding procedure between a PDMS cutout made by soft-lithography and the developed trap substrate to form a sealed microchannel. c) shows a widefield microscope image of a cell trap array inside a microfluidic device captured with a $4 {\times }$ magnification objective. The red lines indicate the borders of the channel. The inset shows a detailed view of the cell traps, captured with a $60 {\times }$ magnification objective. The visible black marks serve as alignment guides for the cell trap array with the channel

The following Section gives an overview of the methods used to produce microfluidic cell trap devices by 2PP and employ them to trap yeast cells for TIRF imaging. In general, the fabrication of cell trap devices involves two major steps: the printing of traps on a coverslip by 2PP (as shown schematically in Fig. 2a)) and the bonding of a PDMS mold to encapsulate the structures in a microfluidic channel (shown schematically in Fig. 2b)). Section 2.3, describes the fabrication steps for producing cell trap devices in detail. This involves the preparation of photoresists, the 2PP printing process, and the soft lithography process to produce PDMS-based microchannels. Section 2.4 provides information on the yeast cells used for trapping experiments. Additionally, the procedure for fabricating samples with immobilized cells is described. Finally, in Section 2.5, the TIRF microscope setup is presented.

### Setup for two-photon polymerization

A custom-built 2PP setup is used to fabricate cell traps on standard coverslips. The writing laser is a pulsed femtosecond laser (Axon 780, *Coherent*) with a central wavelength of 780 nm and a repetition rate of 80 MHz. The beam is expanded $10 \times$ by a telescope and is coupled to the back port of a microscope body, where a dichroic mirror (Multiphoton-Emitter HC 720/SP, *AHF Analysentechnik*) reflects the beam towards the sample. The expanded beam is then focused by a $100{\times }$ magnification objective with a numerical aperture (NA) of 1.45 (Plan Apo $\lambda$ $100{\times }$, *Nikon*). The microscope stage is equipped with a piezo stage (P-545.3C8S0, *Physik Instrumente*), which allows precise movement along all spatial axes in a range of 200 $\upmu$m and a resolution of 1 nm. The printing process can be performed at adjustable speed, with a maximum velocity of 120 $\upmu$m/s for the typical dimensions used in this study. For larger structure arrays exceeding the piezo stage’s range, the sample can be positioned with high precision in the xy-plane using two stepper motors (P-545.USC, *Physik Instrumente*). The writing process is monitored by a CCD camera, whereas the white LED of the microscope is filtered through a yellow bandpass filter to avoid exposing the sample to ultraviolet light. Additionally, a continuous-wave 532 nm laser is coupled to the microscope via the same port as the infrared laser which is used to align the writing plane with the sample surface. This ensures that polymerization starts at the interface between the glass substrate and the photoresist, resulting in structures that adhere to the glass bottom.

### Two-photon polymerization of cell traps

Unless otherwise stated, the photoresist used to create the traps is *Ormocomp*^®^ (*micro resist technology*). First, a drop of photoresist ($\sim 100\, \upmu$l) is cast onto a standard coverslip. The coverslip is then heated for 5 min at 80 °C on a heat plate and later placed in the setup described in Section 2.1. The writing laser is operated at a mean power of 39 mW (measured at the laser output) and the stage is set for a maximum velocity of 100 ${\upmu }$m/s during the fabrication process. Further details on the selection of these values are available in the supplementary material. Cell trap models are first created as standard CAD files and then converted into custom writing scripts for the 2PP setup. Under these conditions, a single cell trap is usually fabricated within (2-5) min, depending on the model used. Most cell trap arrays are created with 25 trap structures per row. The distance between each trap within a row (perpendicular to the flow direction) is equal to the width of the trap structure with 8.4 ${\upmu }$m. Along the flow direction, a larger distance of 15 ${\upmu }$m is chosen to prevent clustering of cells. From row to row, the pattern of the structures is shifted by the width of one trap, to increase the likelihood that a cell passing a row of structures is captured in the subsequent row. After the entire array is completed, the residual photoresist is removed by immersing the sample in developer solution (OrmoDev, *micro resist technology*) and rinsing it multiple times.

The sample preparation for devices containing hydrogel cell traps differs slightly from the previous explanation. In the following, the important differences are summarized. The hydrogel-based photoresist is prepared by mixing 1.5 wt% of the photoinitiator 2-Benzyl-2-(dimethylamino)-4$^\prime$-morpholinbutyrophenon (405647, *Sigma Aldrich*) in Poly (ethylene glycol) diacrylate (PEGDA) with a molecular weight of $M_n=250$ (475629, *Sigma Aldrich*). When devices containing hydrogel cell traps are prepared, the substrates are silanized first to enhance the adhesion of structures to the substrate. For this, coverslips are treated with air plasma for 2 min at RF power equal to 29.6 W. Subsequently, the substrate surface is covered with 3-(Trimethoxysilyl)propyl methacrylate (440159, *Sigma Aldrich*) for 5 min. Afterwards, the remaining silane is removed with an air gun. After a drop of the hydrogel precursor is cast onto the coverslip, the sample is processed as described previously. After the cell traps are fabricated, the remaining precursor is removed by immersing the sample in isopropanol for at least one day.

### Fabrication of microfluidic chips

Microfluidic chips are fabricated by means of standard soft lithography in PDMS. First, a thin layer of SU-8 2010 photoresist (*Microchem*) is spin-coated onto a silicon wafer at 500 rpm for 30 s, followed by 1000 rpm for an additional 30 s. According to product specifications, this process results in an approximate layer thickness of 20 ${\upmu }$m. The wafer is heated at 95 °C for 5 min, followed by exposure to UV light ($\lambda =365$ nm) with an exposure density of 33 mW/cm^2^ for 5 s. Exposure is performed with a photomask (*Micro Litho*) applied to the wafer, ensuring that only the regions forming the channel structure are cured. In this study, we use simple designs of straight channels with a width of 100 ${\upmu }$m, each featuring a single inlet and outlet. After UV exposure, the wafer is baked again at 65 °C for 1 min, followed by 95 °C for 5 min. The uncured SU-8 is then removed using SU-8 developer (*Microchem*).

Microfluidic chips are fabricated from the finalized wafers using replica molding. To do this, 50 g of PDMS is mixed with 5 g of curing agent (Sylgard 184, *Mavom*) and degassed in a vacuum chamber for 1 h. The mixture is then poured into the mold and baked at 80 °C for 2 h. Afterwards, the solidified PDMS is peeled off the wafer, and the respective channel structures are cut. Inlet and outlet holes are punched using a biopsy punch tool. Both the resulting PDMS blocks and standard coverslips are cleaned with isopropanol. Before bonding the PDMS to the sample substrate, the position of the cell trap array on the coverslip is marked with a marker to aid in alignment. Subsequently, the prepared coverslip and PDMS channel are treated with air plasma for 2 min at an RF power of 29.6 W. Immediately afterward, the PDMS and coverslip are permanently bonded by placing the plasma-activated surfaces together and applying gentle pressure. The coverslip is placed on a millimeter-scale grid to ensure proper alignment of the channel with the cell traps. A final channel height of $(20 \pm 2)\,{\upmu }$m is measured during the preparation of final channels, by evaluating the range in which polymerization can occur. A wide-field microscope image of the completed cell trap device is shown in Fig. 2c).

### Cell culture and preparation of cell samples

The yeast cell strains used in this study are derived from the Saccharomyces cerevisiae BY4741 MATa strain (*Euroscarf*). Genomic tagging of the plasma membrane protein Pma1 with GFP is performed using standard procedures published elsewhere (Janke et al. 2004). Cell cultures are grown overnight in yeast extract peptone dextrose (YPD) medium at 30 °C, washed three times with H_2_O and diluted with synthetic complete media without methionine to an $\text {OD}_{600} = 3.1$. Subsequently, cells are further grown for 2.5 h at 30 °C. For experiments with fixed cells, cell solution is dispersed on coverslips coated with 1 mg/mL concanavalin A (*Sigma Aldrich*).

To prepare cell solution for microfluidic experiments, the initial solution is diluted with SC-Ura (synthetic complete media without uracil) to achieve the desired concentration of cells. Two methods to inject solution in microfluidic devices are used in this study. In the first one utilizing a syringe pump, the microfluidic device is fixed in the TIRF microscope setup explained in Section 2.5 and the inlet of the device is connected to a reservoir of cell solution. The outlet is connected to the syringe pump containing an empty syringe, which is slowly drawn up to suck solution inside the device. This procedure has the advantage of reduced contamination inside the device compared to just injecting solution with a syringe from the device’s inlet. In the second injection method, a pipette is used. The pipette tip is inserted directly into the inlet of the microfluidic device in the TIRF setup, and approximately 50 ${\upmu }$L of solution is injected instantly.

### TIRF imaging setup

Epifluorescence and TIRF microscopy are performed on a fully automated IMIC stand (*FEI/Till Photonics*) using a diode pumped solid state laser at 491 nm (*Coherent Sapphire*). The incidence angle of the excitation laser is adjusted using a two-axis scan head. For TIRF imaging, a $100 {\times }$ oil immersion objective (*Olympus*) with an NA of 1.45 is used. Image acquisition is performed using an iXON DU-897 EMCCD camera (*Andor*) and Live Acquisition (*Till Photonics*) software.

## Results and discussion

The following section presents the results from experiments with microfluidic devices containing cell traps made by 2PP. First, in Section 3.1, two different base designs of cell traps are tested and compared with respect to their selectivity for single cells, the fixation of trapped cells, and the image quality they produce when performing TIRF microscopy. The superior design is further investigated in Section 3.2, where the results for different configurations of the structural dimensions are shown and discussed. Here, special focus is placed on the height of the cell traps and its impact on trapping efficiency, as this parameter is especially difficult to adjust precisely. Subsequently, the trapping efficiency of the optimized cell trap design based on the cell concentration *c* and the injection method is measured in Section 3.3. The potential of the fabricated device for TIRF imaging is further evaluated in Section 3.4, where the results are compared to conventional cell immobilization methods. As a final step, a similar microfluidic device is fabricated, whereas the conventional photoresist for producing the trap structures is replaced with a custom hydrogel-based photoresist. The results for cell trapping and TIRF imaging using this device are shown in Section 3.5.

### Comparison of different cell trap designs

**Fig. 3:**
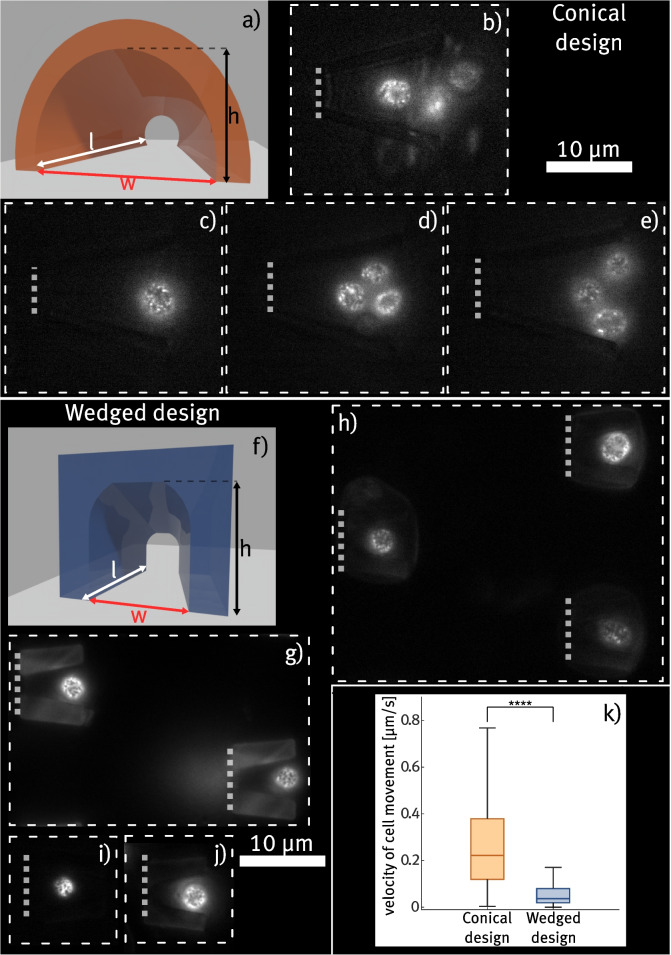
Comparison of the conical cell trap design and the wedged design. a) and f) shows the 3D models of the designs with labeled dimensions, respectively. b)-e) shows exemplary TIRF images of trapped cells in conical cell traps, in which multiple cells get trapped more often. g)-j) shows recordings from wedged cell traps. Each visible cell is trapped in a separate cell trap structure here. In k), the measured mean velocities for both cell trap designs are compared. Velocities are determined by automatically tracking trapped cells and determining the lateral motion in between each frame. Gray dashed lines in the microscope images indicate the rear opening of cell traps. The membrane protein Pma1 of all shown yeast cells is tagged with GFP, facilitating the visualization of the cell membrane through TIRF imaging

For the first experiment, devices containing cell traps with a *conical* shape are fabricated. An illustration of the basic shape with labeled dimensions can be seen in Fig. 3a). The basic idea of this design is that a yeast cell entering the trap would eventually get stuck in the increasingly narrow cone, thus blocking the flow through the cell trap so that no further cells are caught in the same trap. Still, the liquid flowing along the trap containing an immobilized cell exerts pressure on the cell, keeping it inside the trap. The height gradient within the trap ensures that all yeast cells, regardless of potential size differences, are effectively trapped and securely fixed in contact with the glass substrate. In a first step, a test sample containing *conical cell traps* with different dimension configurations is printed. The opening width *w* is varied in a range of $(10-12)\,{\upmu }$m, the opening height *h* in a range of $(7-9)\,{\upmu }$m and the trap length in a range of $(14-16)\,{\upmu }$m. For the opening on the rear side of the trap, sizes between $(3-4)\,{\upmu }$m for both width and height are chosen, which should be smaller than the diameter of the yeast cells (which is approximately $(4-6)\,{\upmu }$m Sherman 1991) and therefore prevent them from passing through the traps. The prepared device is tested by injecting a solution of yeast cells (with $c\approx 130\,{\upmu \text {L}^{-1}}$) at a constant flow rate of 5 ${\upmu }$L/min. Simultaneously, the interiors of the cell traps are examined using TIRF microscopy.

Figure 3b)-e) show that the structures are capable of trapping cells and enabling the visualization of protein structures at the cell membrane. This indicates that those cells are in direct vicinity to the substrate surface and proper TIRF imaging is enabled. However, most cell traps remain empty ($\sim 79{\%}$) even after constantly pumping the cell solution for 2 h or catching more than one cell inside ($\sim 10{\%}$), as can be seen in Fig. 3d)-e). This happens either if cell clusters are trapped, or when cells are trapped consecutively as a residual flow through the trap remains after the first cell enters. The remaining flow inside filled traps can also be inferred from the strong motion of trapped cells. Even cells that are stuck at the narrowest part of the trap show visible translatory motion, rotation, or jittering for all different trap dimensions. Examples of cells displaying significant motion in the flow can be observed in Video 1, provided in the supplementary material. This also greatly impacts the potential to record high-quality TIRF images with larger integration times.

Based on these results, a second cell trap design is created in which the inner space becomes narrower in a *wedged shape*, as can be seen in Fig. 3f). As the cross-section of this cell trap’s opening is less circular compared to the conical cell trap, it is expected that more residual flow is maintained inside the trap, even when a cell is already present. Therefore, the overall size of the trap is designed to be much smaller, allowing it to hold only a single cell. The residual flow within the trap may enhance the stability of the cell trapping, as the cell experiences a constant flow inside. Again, test devices containing cell traps with different size configurations are prepared to evaluate the feasibility of TIRF imaging. To prevent cell clusters from entering, the entry width is chosen comparatively narrow with a range of $(5-6)\,{\upmu }$m. The length *l* is also chosen to be shorter in the range of $(7-8)\,{\upmu }$m, and the height *h* is varied in the range of $(6-8)\,{\upmu }$m. More information on how these values were chosen can be found in the supplementary material. Similar to the previous trap configuration, microfluidic chips containing arrays of cell traps with a certain size configuration are tested by injecting cell solution while performing TIRF imaging. Figure 3g)-j) show exemplary TIRF images of trapped cells inside wedge-shaped structures. For all sizes tested, the number of traps containing single cells is largely enhanced. This also allows to image the cells in neighboring traps simultaneously, as can be seen in Fig. 3g) and h). A quantitative analysis of the trapping efficiency is given in Section 3.3.

Using visual observation, we are able to verify that the adapted trap design largely reduces the motion of cells inside the structure even at constant flow inside the channel. Representative recordings of trapped cells with reduced motion within the structures are shown in Video 2, available in the supplementary material. To determine the motion quantitatively, time lapses of trapped cells are captured for both the conical and wedge-shaped trap designs in the normal epifluorescence configuration. From these videos, the total lateral motion (along the x- and y-axis, respectively) between each frame is extracted and divided by the integration time for each frame, which differs from ($0.15-1$) s depending on the intensity of the signals. By that, the mean absolute velocity of cell motion is calculated. Figure 3k) shows a comparison of the values determined for both designs. The graph demonstrates that motion within wedge-shaped traps is significantly reduced, with a mean velocity of $(0.07\pm 0.08)\,{\upmu }$m/s, compared to the conical design, which exhibits a mean velocity of $(0.35\pm 0.30)\,{\upmu }$m/s. By estimating the diffusion coefficient of a yeast cell using the Stokes-Einstein-Sutherland equation (Sutherland 1905), the expected displacement of a yeast cell undergoing Brownian motion is approximately 0.2 ${\upmu }$m per second. This suggests that the wedge-shaped trap effectively minimizes cell fluctuations, providing a notable improvement in stabilizing the cells. This observation can also be confirmed when the syringe pump flow is deactivated. In the case of the conical traps, many cells escape due to minor backflow or Brownian motion. In contrast, most cells remain securely in place within the wedge-shaped traps even in the absence of continuous flow. This suggests that the smaller opening size of the wedge-shaped traps provides additional mechanical stability to the trapped cells, ensuring they are not solely reliant on flow to stay in place. Still, cells can be removed from the wedged cell traps by applying flow in the opposite direction using the connected syringe pump. The reduced motion of the investigated specimen also results in enhanced quality of the TIRF images. Especially the recordings explicitly optimized to image a specific cell, shown in Fig. 3i) and j), demonstrate promising results even without additional image processing, aside from enhancing the contrast. The imaged protein patterns are less noisy than the comparable recordings from the conical traps. Here, the contrast between the fluorescence signal from the cells and the cell trap structures is particularly prominent. This is investigated further in Section 3.4.

### Optimization of cell trap dimensions

As the wedged cell trap design emerged as favorable in Section 3.1, the dimensions of this design are further optimized to provide the maximum efficiency for cell trapping. From the experiments discussed in Section 3.1, it is found that cells are successfully trapped regardless of the chosen entry width *w*. For widths larger than 5 ${\upmu }$m, trapped cells tend to enter further into the cell trap, which results in free space at the entrance and could eventually allow a second cell to be trapped. To avoid this, the width of 5 ${\upmu }$m is set for the final configuration, which should be close to the feasible minimum according to the mean diameter of the used cells with $(4-6)\,{\upmu }$m (Sherman 1991). The effectiveness of different cell trap lengths *l* is tested using silica beads with a diameter of 5 ${\upmu }$m. The fixation of beads trapped inside the structures is tested by trying to move the beads out by means of optical tweezers using experimental setup published elsewhere (Kumar et al. 2023) (see supplementary material for more details). From this, it is found that the length does not seem to have a major influence on how far the beads reach inside the trap and how much force is needed to remove them again. Beyond that, the length of the cell trap influences the contact area of the whole structure with the substrate. For structures with high aspect ratios that experience stress in the flow of the microchannel, a too small contact area between the structure and the substrate can lead to detaching of the structure if the adhesion of the photoresist is not high enough. In all experiments presented here, it is found that no structure detaches from the substrate even if exposed to high flow rates of up to 1 mL/min. Thus, using the smallest tested dimension of 7 ${\upmu }$m presents no disadvantages and is favorable as it reduces the printing time for each cell trap.

**Fig. 4:**
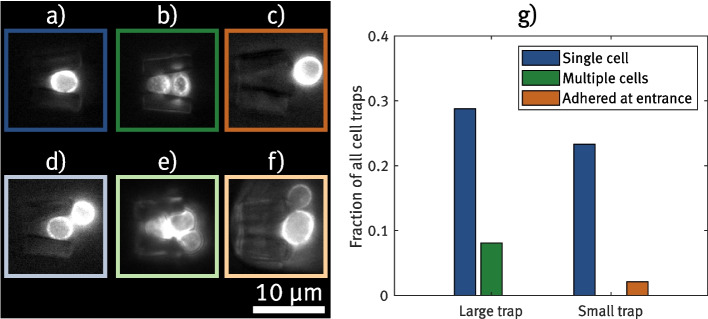
Comparison of different trapping cases. a)-f) show example images for each case distinguished in this measurement here and the trapping efficiency measurement (cf. Fig. 6). a) shows a single trapped cell. b) shows two trapped cells. c) shows an empty cell trap with a cell adhering at the entrance. d) shows a single trapped cell, with another cell adhering at the entrance. e) shows two trapped cells, with another cell adhering at the entrance. f) shows an empty cell trap with multiple cells adhering at the entrance. The membrane protein Pma1 of all shown yeast cells is tagged with GFP, facilitating the visualization of the cell membrane through TIRF imaging. g) shows a statistical comparison of immobilized cells in cell trap arrays, once with an opening height *h* of 7 ${\upmu }$m and once with an opening height of 6 ${\upmu }$m. For each cell trap in the respective array, it is determined whether it contains exactly one cell, multiple cells, or a cell that adhered at the entrance without fully entering the trap

Optimizing the entry height *h* requires more detailed examination compared to the other two dimensions, as deviations from the intended writing path can be more pronounced in the dimension perpendicular to the substrate. This can be derived from Abbe’s diffraction limit by which the resolution of 2PP is naturally lower in the axial direction (along the axis of the printing laser) than in the lateral directions (Jaiswal et al. 2023). Consequently, the value for *h* is already chosen higher than the corresponding opening width *w*, even if the cell shape is expected to be approximately circular. To determine which value of *h* yields a good result for trapping efficiency, two cell trap devices are fabricated with arrays of traps similar to the layout shown in Fig. 2c). The trap configuration is the same for both devices, except that *h* is set at 6 ${\upmu }$m for one device and 7 ${\upmu }$m for the other device. For both devices, the yeast cell solution at $c\approx 5 \cdot 10^3\,{\upmu \text {L}^{-1}}$ is injected using a micropump at a constant flow rate of 10 ${\upmu }$L/min. As soon as the solution reaches the traps, the flow is maintained for 10 min and then stopped. The number of filled cell traps is determined by examining their interiors using fluorescence microscopy. For each trap structure, it is distinguished whether a single cell is trapped, multiple cells are trapped, or if a cell adhered at the entrance of the structure but did not end up inside the trap. Examples of all three cases can be seen in Fig. 4a)-c).

Figure 4g) shows which percentage of the total cell traps exhibits one of the three cases for both tested devices. In direct comparison, the cell trap array with an opening height of 7 ${\upmu }$m shows a higher fraction of traps containing a single cell at 29% compared to 23% for the traps with an opening height of 6 ${\upmu }$m. Furthermore, roughly 8% of the traps with the larger openings contain two cells, whereas none are found in the array with the smaller openings. In contrast, approximately 2% of the traps have a cell adhering at the opening without entering here. Although the percentage is comparably low, it is observed that cells adhering to the trap entrance can cluster together with other passing cells, which can lead to clogging of the respective area in the array. To mitigate this issue, and given the previously demonstrated more effective single-cell trapping, the larger opening height of 7 ${\upmu }$m is considered the better choice for future experiments. The device with the dimensions specified in this section is further tested in terms of trapping efficiency in Section 3.3. Additionally, it should be noted that despite the differing trapping efficiencies, no differences in the stability of trapped cells were observed between the two designs, as evidenced by the absence of cells escaping the traps. Thus, the smaller empty space within the cell trap with a 6 ${\upmu }$m opening, which might suggest reduced residual flow, does not appear to negatively impact the force holding the cell in place. This supports the hypothesis that cells are mechanically clamped within the wedged cell traps, with the flow playing a less significant role in retaining the cells compared to the conical cell traps.

### Investigation of trapping efficiency

**Fig. 5:**
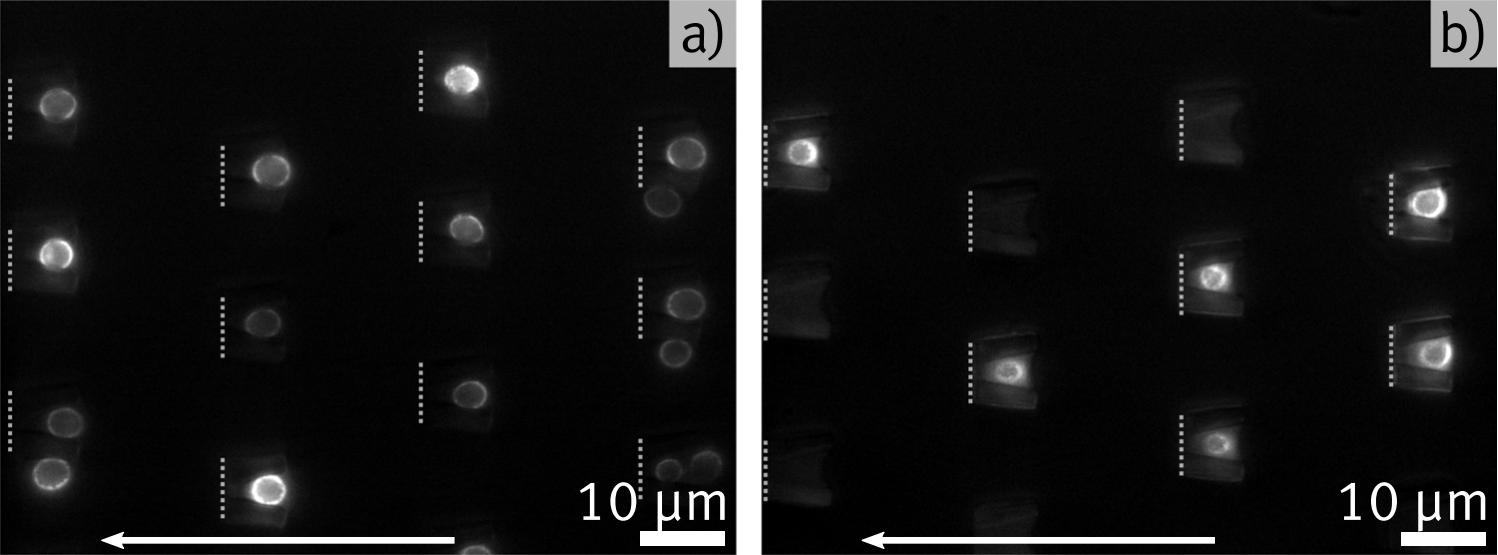
Overview of cell trap arrays imaged by fluorescence microscopy. a) shows an array filled with a high cell concentration *c*. All 12 traps visible in the frame are filled with a single cell, but there are also multiple cells adhering to the outside of the structures. b) shows an equal array, but here, a solution with a lower concentration *c* is injected. Not all of the traps visible in the frame contain a cell, but there are also no cells immobilized outside the structures. Gray dashed lines in the images indicate the rear opening of cell traps. The membrane protein Pma1 of all shown yeast cells is tagged with GFP, facilitating the visualization of the cell membrane through TIRF imaging

Following the findings in Section 3.2, a cell trap design with an opening width *w* of 5 ${\upmu }$m, a length *l* of 7 ${\upmu }$m, and an opening height *h* of 7 ${\upmu }$m is determined as optimal. In the following, cell trap arrays containing 16 rows with 25 traps per row are tested regarding their trapping efficiency. Similarly to Section 3.2, cell solution with $c\approx 5 \cdot 10^3\,{\upmu \text {L}^{-1}}$ is injected into the trap array using a micropump. The space inside the traps is again investigated using fluorescence microscopy, whereas here the six cases shown in Fig. 4a)-f) are distinguished. This includes the case in d), where a single cell is trapped, but another one adheres at the entrance, the case in e) where multiple cells are trapped and adhere at the entrance and the case that more than one cell adhered at the entrance without a cell being inside the trap in f). Subsequently, this measurement is repeated with another microchannel containing the same cell trap array, but this time the cells are injected with a pipette, as described in Section 2.4, in contrast to the syringe pump method. The findings in Section 3.1 indicate that the wedge-shaped cell traps do not rely on a constant flow of liquid to ensure reliable trapping, making it possible to achieve effective trapping with a single injection. As a final step, the measurement is repeated twice using both injection methods, respectively, but here a higher cell concentration of $c \approx 5 \cdot 10^6\,{\upmu \text {L}^{-1}}$ is used to investigate the impact on trapping efficiency. In Fig. 5, two overview pictures of trapped cells are shown in this experiment, where a) is captured after injecting the solution with the higher concentration. It shows that all 12 cell traps in the image contain a single cell, with 4 cells adhering to the outside of the traps in total. Figure 5b) shows an example at low concentration with 6 out of 12 traps being filled successfully, but without cells adhering to the outside.

**Fig. 6:**
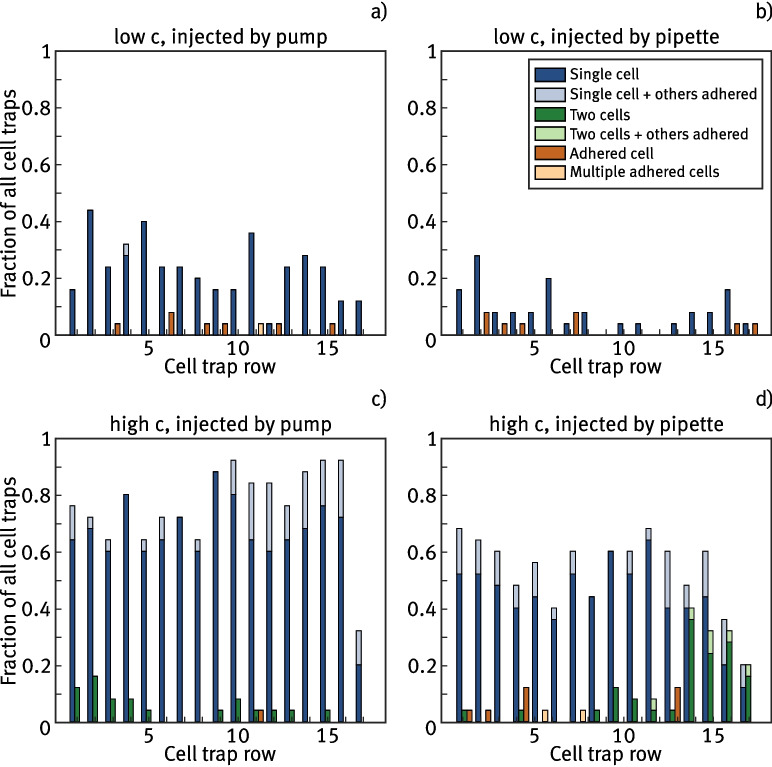
Statistical results for trapping efficiency experiments, in which cell solution is injected in microchannels containing cell trap arrays. Consequently, each cell trap is examined and categorized based on the cases presented in Fig. 4. Each plot shows the ratio for the respective cases along the 16 cell trap rows of the tested device. The results differ based on different initial cell concentrations and the method used to inject the cells. The y-axis is the same for all subfigures

Figure 6 shows the distribution of different trapping cases for each of the described measurements. In each case, the x-axis represents the number of rows of the cell trap array, while the y-axis shows the fraction of cases specified in Fig. 4a)-f) in the respective row. The single pictures in Fig. 4 are labeled with the corresponding bar color in Fig. 6. Generally, the results indicate that a higher cell concentration yields more trapped cells, but at the expense of more cells adhering at the entrance of the filled cell traps or multiple cells getting trapped. This result seems intuitive, especially considering that at a higher concentration, the cells tend to form clusters that occasionally get stuck at the array. When comparing the results of using a syringe pump versus a single pipette injection, the overall amount of trapped cells is higher for the cases in Fig. 6a) and c) where the syringe pump is used. However, the fractions of cell traps containing adhered or multiple trapped cells do not appear to decrease uniformly when using the syringe for injection. In fact, at high cell concentrations, a relatively large number of multiple trapped cells is observed in the last four rows. One possible explanation is that pipette injection likely induces a more unstable flow within the channel compared to the steady flow generated by the syringe pump. Consequently, cells may experience stronger localized flows, leading to increased pressure that forces cells more firmly into the traps at certain positions. Additionally, it is conceivable that the distribution of cells within the solution varies between the two injection methods. The slow, steady flow from the syringe pump might reduce cell clustering, whereas pipette injection may result in more cell clusters, leading to a higher number of multiple trapped or adhered cells. Moreover, occupied cell traps in the front rows of the array might create a screening effect, increasing the pressure and flow velocity in the remaining free spaces of the channel. This could contribute to a higher occurrence of multiple trapped cells toward the rear of the array. Nevertheless, the trapping efficiency for single cells remains high with (40-70) % throughout the first 14 rows, suggesting that single pipette injection is a feasible method for utilizing the developed device. For high cell concentrations, the trapping efficiency exceeds 90 % throughout the channel when using pump-based injection. In general, trapping is more effective at the front and back of the array, with a noticeable dip in single-cell trapping efficiency in the middle, which is observed in all four cases. This effect is particularly pronounced at lower concentrations and when using the micro pump in b). A possible explanation based on Bernoulli’s equation is that changes in static pressure at the front and back of the array generate net forces that affect trapping efficiency. To further enhance the device, future experiments could focus on optimizing the flow within the channel to achieve uniform trapping across the entire array. This could be achieved through numerical simulations of the fluid flow around the cell trap array.

Considering all the cell traps in the entire array, the configuration shown in Fig. 6b) exhibits the lowest overall efficiency, with only 8.7 % of the traps containing one or more cells. In contrast, the case shown in Fig. 6c), using a high cell concentration and pump injection, results in an overall filling ratio of 80.5 % for the traps.

**Fig. 7:**
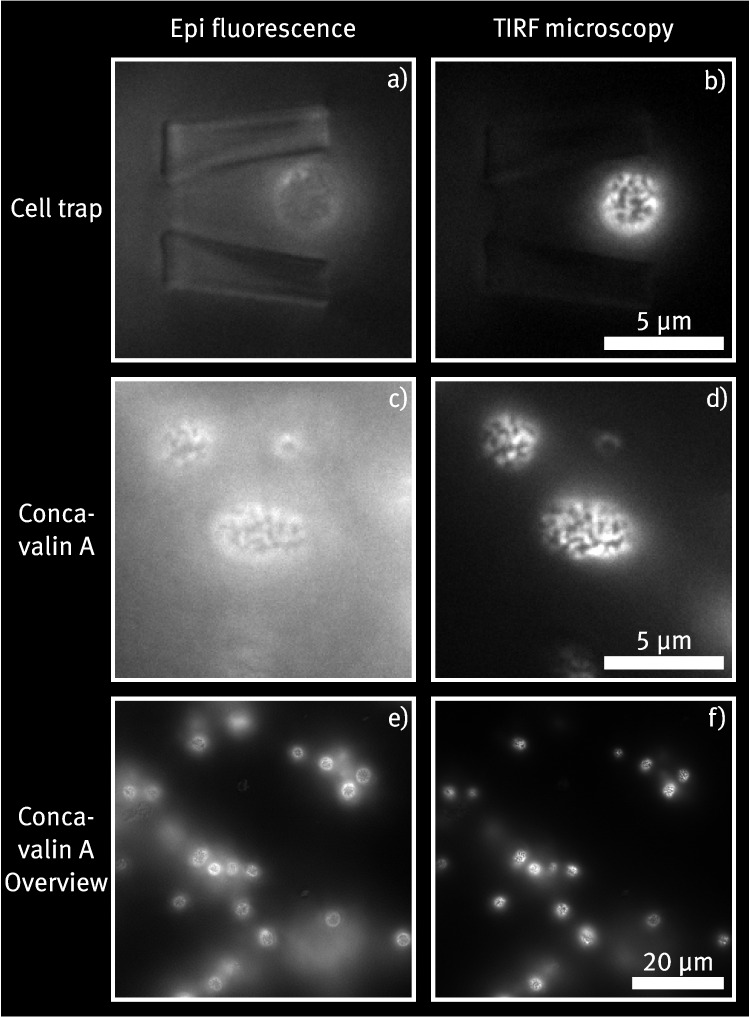
Comparison of immobilized cells using Concanavalin A and custom cell traps. In all cases, cells are imaged using epifluorescence (left side) and optimized for imaging the cell membrane with TIRF (right side). a) and b) shows a single cell captured in a cell trap made by 2PP. c) and d) show detailed images of a cell immobilized on a coverslip using Concanavalin A. e) and f) show overview images of a cell cluster on a coverslip using Concanavalin A. The membrane protein Pma1 of all shown yeast cells is tagged with GFP, facilitating the visualization of the cell membrane through TIRF imaging

It is important to note that the trapping efficiency is expected to be influenced by the number of cells injected into the device. This depends on factors such as the cell concentration in the solution, the flow rate, and the duration of injection. Although efforts are made to maintain consistent injection times and flow rates during the pump injection procedure, unstable flow is induced particularly when connecting the tubes to the microfluidic device and starting the flow. Even after allowing several minutes for the flow to stabilize, fluctuations in the flow rate cannot be entirely eliminated. To better assess global trapping efficiency in relation to the total number of injected cells in future studies, longer injection procedures with significantly lower cell concentrations should be considered. Achieving sufficient results may require larger cell trap arrays, which could be facilitated by enhancing the used printing setup. Recent advancements have demonstrated that 2PP can achieve fabrication speeds of several millimeters per second, surpassing the speeds achieved in this study by several orders of magnitude (Jaiswal et al. 2023). These advancements have been significantly driven by progress in the commercial development of 2PP technology in recent years. Additionally, innovative strategies such as employing multiple foci during printing hold great promise for further accelerating the fabrication of 2PP structure arrays (Geng et al. 2019; Plidschun et al. 2022), thereby making the presented approach suitable for studies requiring high-throughput cell analysis.

### Comparison of TIRF imaging with conventional methods

In the following, the device specified in Section 3.2 is investigated further with respect to the TIRF image quality of trapped cells. Figure 7a) and b) show exemplary trapped cells, captured once with standard epifluorescence in Fig. 7a) and once under TIRF imaging conditions in b). In the epifluorescence picture, the cell trap structure is clearly visible due to autofluorescence of the Ormocomp^®^ photoresist. Conversely, the outlines are barely visible in the TIRF image, indicating that the autofluorescence signal can be significantly reduced by properly adjusting the incident angle of the excitation light. The protein structures on the cell membrane are clearly resolved, even without applying deconvolution on the images.

**Fig. 8:**
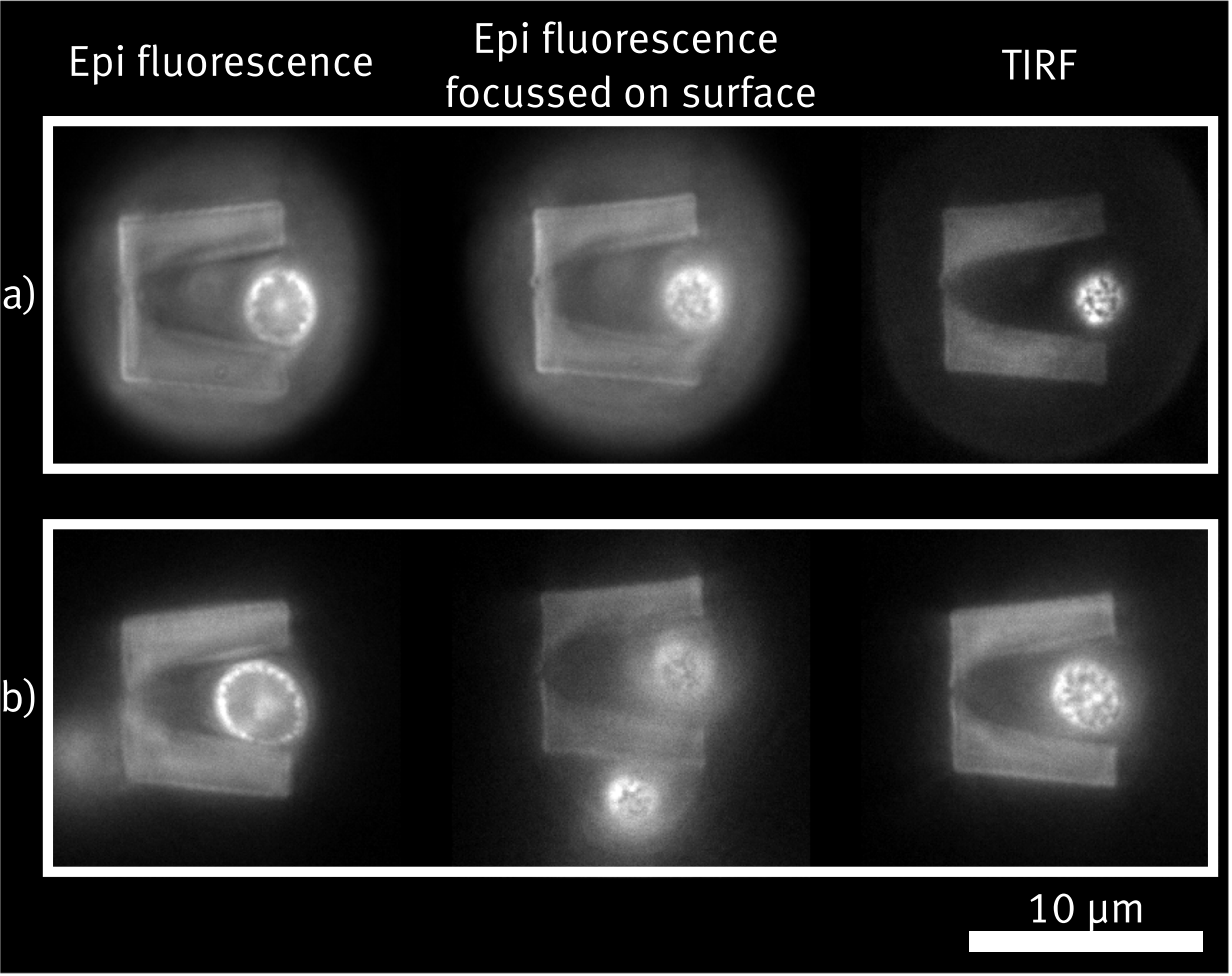
a) and b) show two examples of single cells immobilized in a trap made from PEGDA hydrogel. The leftmost images show epifluorescence images focused on the middle of the cells to visualize where the cell connects with the structure. The middle images show epifluorescence images with the focus set on the cell membrane adjacent to the substrate. The rightmost images show the same focus plane, but with the imaging angle optimized for TIRF imaging. The membrane protein Pma1 of all shown yeast cells is tagged with GFP, facilitating the visualization of the cell membrane through TIRF imaging

To compare TIRF image quality, a sample of the same cell culture used in Section 3.3 is immobilized on a coverslip using Concanavalin A and imaged directly after using the TIRF setup. Figure 7c) and d) show detailed images of one cell fixed this way, while Fig. 7e) and f) show an overview over a immobilized cell cluster, captured with epifluorescence and TIRF configuration in both cases. Because cells simply adhere randomly to the surface, their distribution on the sample is rather inhomogeneous. In fact, the cells seem to cluster together here, as can be seen especially in the epifluorescence images. As a result, even with contrast adjustments, blurs from cells outside the image plane remain visible. This may impede image quality and hinder processing steps, such as thresholding to automatically detect cells in the image. When comparing the TIRF images of the cells immobilized on the coverslip with those of trapped cells in Figs. 4 and 7b), the cells on the coverslip generally exhibit a more elongated shape, while most of the trapped cells appear round. This is especially prominent in the magnified images in Fig. 7c) and d). An elongated shape might indicate cell stress, e.g. due to starvation or environmental stress. In this context, the trapping device offers the advantage of continuously replenishing the media surrounding the immobilized cells, potentially improving their physiological conditions and enabling longer imaging durations. In contrast, samples prepared with concanavalin A must be imaged within approximately one hour after preparation due to gradual dehydration. In addition, the elongated shape of the cells could also be caused by a smaller contact area. Because the cells are not pressed onto the substrate like the trapped cells and only adhere due to contact with the applied protein, conceivably, a generally smaller fraction of the cell surface is in direct contact with the glass. Consequently, this fraction appears smaller in the focal plane imaged by the TIRF microscope. In contrast, when comparing epi-fluorescence and TIRF images of trapped cells, such as those shown in Fig. 7a) and b), the cells appear mostly round and of similar size. Given the spherical shape of yeast cells (Feldmann 2012), one would expect the TIRF images to show a relatively small area, as only the portion of the cell membrane in direct contact with the glass substrate would be excited. The fact that the TIRF images reveal a larger cross-section of the membrane suggests that the yeast cells are being pressed onto the coverslip within the cell trap, bringing a significant portion of the membrane into close proximity to the substrate. This observation highlights the potential of the presented approach for imaging cells that are otherwise challenging to visualize using TIRF microscopy due to their mechanical properties or shape, as it enables to visualize a larger fraction of the entire cell membrane.

### Testing hydrogel-based cell trap devices

As a proof of concept, we create the same cell trap device presented in the previous sections, but instead of the commercially available photoresist Ormocomp^®^, we use a PEGDA-based hydrogel precursor to create the microstructures. The potential to print cell traps with different properties such as biocompatibility, mechanical features, or functionalizability can extend the possible applications of the device.

The experimental design is similar to the one presented in Sections 3.2 and 3.3. A cell solution with $c \approx 5 \cdot 10^3\,{\upmu \text {L}^{-1}}$ is injected inside the channel via a syringe pump at a set flow rate of 10 ${\upmu }$L/min. Figure 8 shows two exemplary trapped cells. It is observed that cells get trapped inside the hydrogel-based cell traps, though they generally penetrate less deep compared to the Ormocomp^®^ traps. From the epifluorescence, it becomes evident that the cells are not touching both sides of the trap as is usually seen in the traps made of Ormocomp. A possible conclusion from this is that the motion of the cells is blocked in the z-direction, resulting in the cells being primarily quenched in between the top layer of the cell trap and the glass substrate. As a result, it is observed that the trapping efficiency of the cells is not as high as that of the traps made from Ormocomp, so that eventually only few cell traps contain a cell. There are multiple explanations for deviations from the original design. First, using a different photoresist requires a different laser power to be used during the 2PP process. This is because each photoresist has other laser dosages at which it can be polymerized efficiently and at which thermal damages occur (Zandrini et al. 2019). As already briefly discussed in Section 3.2, the printing resolution in x-, and y-directions differ from the one in z-direction as a result of the shaping of the printing laser focus. It is also expected that they do not show the same dependency on the excitation laser power, hence, the final structure might deviate slightly when using different photoresists and different laser powers. Secondly, hydrogels tend to swell when exposed to aqueous media, such as the cell medium (Kopeček 2007). The used PEGDA precursor has a comparatively low molecular weight, which should yield a lower degree of swelling, and before the experiment, the sample is not immersed in water. Although during the course of the experiment (usually up to 2 h per channel) no visible changes of the traps caused by swelling can be observed, it is still likely that the dimensions of the structures change slightly. Again, it is not clear whether the degree of swelling is equal in all spatial directions, as structural changes caused by inhomogeneous light exposure during fabrication might have an effect on swelling behavior. In future experiments, this could be clarified with an extensive investigation of the structure dimensions by, e.g., scanning electron microscopy.

For cells that are successfully trapped, the potential for TIRF imaging is again investigated. Figure 8 shows the respective TIRF images for the selected examples in the right column. It can be seen that the protein structures of the cells can be resolved similar to the results in Section 3.4. A key difference to the traps made from Ormocomp^®^ becomes evident when comparing the TIRF images in Fig. 8 with those in Fig. 7a) and b). Apparently, the structures made from PEGDA exhibit much stronger autofluorescence and therefore appear much brighter in the images. This could reduce image quality for the investigated cells if their signal overlaps with that emitted by the cell traps. Furthermore, this circumstance impedes the automation of cell detection or analysis. For example, applying an intensity threshold to track cells, as done in Section 3.1, would not be as easy here. Nevertheless, the experiment shows that it is feasible to produce cell trap devices from different materials and use them for TIRF imaging. This allows tailoring of the material properties to the specific application, such as the type of cell to be trapped or whether additional functionalization is required. In future experiments, finding stable, biocompatible polymers suited for this application that exhibit lower autofluorescence could be an important step in expanding the application field for the cell trap device.

## Conclusions

We present a new microfluidic device fabricated by a combination of standard soft lithography and 2PP to enable the fixation of yeast cells for TIRF imaging of the cell membrane. We successfully incorporate arrays of microstructures with different designs in conventional PDMS channels. The different designs of cell traps are compared by analyzing the motion of trapped yeast cells inside the structure, showing that a wedged design with flat side walls yields the most effective fixation of cells. The dimensions of the trap structure are further optimized by assessing the trapping efficiency of differently scaled traps. For optimized designs, the impact of cell concentration on trapping efficiency is measured for two injection methods. When injecting a low cell concentration with a syringe pump, the device selectively traps single cells with trapping efficiencies of approximately 40 % for some rows of cell traps. At a higher concentration, trapping of multiple cells occurs occasionally, but the trapping efficiency for single cells reaches as high as 90 % for several rows. Although the trapping efficiency is generally lower, a single injection with a pipette is also performed successfully, which can be useful to reduce the experimental effort required to carry out trapping experiments.

We successfully demonstrate how the height gradient within the cell traps enables the fixation of cells in close proximity to the substrate, regardless of the size of the trapped cells. This feature highlights the advantages of 2PP over standard PDMS-based trapping devices, where the ceiling height of the trapping site is fixed, even when adapted to cell size (Di Carlo et al. 2006; Kobel et al. 2010), potentially leading to instability for smaller trapped cells. The enhanced stability of the trapped cells in our design is demonstrated by imaging of the tagged cell membranes using TIRF microscopy. By optimizing the imaging configurations, autofluorescence from the cell trap structures is minimized, enabling high-contrast investigation of patterns on the cell membrane. In future experiments, the contact surface between the trapping structure and the substrate could be decreased to reduce the autofluorescence signal even further. A direct comparison with conventional immobilization methods shows that the use of the cell trap array poses several advantages, in particular the continuous flow of the medium which allows for longer imaging times of healthy cells and reduces cell clustering. The random fixation of cells using binding proteins here seems to promote the accumulations of cell clusters, resulting in significant background signal even under optimized TIRF conditions. In addition, using the cell trap array offers the advantage of catching cells at certain positions and fixed distances from one another, which could be used in future experiments to automate the imaging procedure. This is not easily possible with standard fixation methods, as here the distribution of cells is mostly random.

As a proof of concept, we showcase that similar results can be achieved using soft PEGDA-based hydrogels as the base material for cell traps. Although those structures exhibit stronger autofluorescence in the experiment, they are also feasible for TIRF imaging on trapped cells and to resolve surface protein patterns. In future experiments, using photoresists that support precise biofunctionalization could offer significant advantages. Given the high trapping efficiency demonstrated in this work, this approach could open up numerous new applications, such as enabling direct contact between cells and functional groups introduced on the surface of the trap structures. Furthermore, using a photoresist that is less viscous than Ormocomp^®^ offers the opportunity to print 2PP structures directly inside the channel and remove the remaining photoresist after the printing step (Barwig et al. 2024; Qian et al. 2023). This could enable the combination of trap structures with more complex PDMS channel designs in the future, for example, to shape the flow inside the channel or simply to enhance the precision of the alignment between channel and microstructures. Moreover, the presented method of integrating cell traps fabricated by 2PP could potentially enhance comparable studies where microfluidic cell trapping is used for size-based cell sorting or for separating budding yeast cells from their daughter cells (Xu et al. 2021; Zhu et al. 2021). Building on the demonstrated advantage of 2PP in this study, enabling cell trapping in all spatial directions regardless of cell diameter, such applications could be further extended to specifically trap mother and daughter cells in two consecutive traps. This setup could facilitate targeted, time-resolved analyses, providing valuable insights into cell development and dynamics.

## Supplementary information

Supplementary Information is available for this paper.

## Supplementary Information

Below is the link to the electronic supplementary material.

Supplementary file 1 (avi 13149 KB)

Supplementary file 2 (avi 2356 KB)

Supplementary file 3 (avi 193576 KB)

Supplementary file 4 (pdf 2307 KB)

## Data Availability

Data used in this study will be published at the repository of the University of Münster “datastore.” under the 10.17879/15928506556
