# Supplementary material for: Lab-on-a-chip device for microfluidic trapping and TIRF imaging of single cells
Source: Biomed Microdevices. 2025 Mar 14;27(1):12. doi: 10.1007/s10544-025-00739-0 (PMC11909039; doi:10.1007/s10544-025-00739-0)
Supplement: Supplementary file 4 — (pdf 2307 KB) [file 10544_2025_739_MOESM4_ESM.pdf]

Lab-on-a-chip device for microfluidic trapping and TIRF imaging of single cells - Supplementary material

Dustin Dzikonski, Riccardo Zamboni, Aniket Bandyopadhyay, Deepthi Paul, Roland Wedlich-Söldner, Cornelia Denz, Jörg Imbrock

1. DETERMINATION OF WRITING PARAMETERS

To optimize cell trap fabrication, various writing parameters were tested, focusing on the laser power (p) and stage velocity (v) during the printing process. These parameters directly influence the laser dose, i.e., the number of photons interacting with the photoresist within a given time. A laser dose below the threshold for two-photon absorption results in incomplete polymerization, leading to unstable structures. Conversely, an excessive laser dose can cause microexplosions during printing, damaging the structures. Generally, higher laser doses result in larger printing voxels, polymerizing a greater volume of photoresist. Thus, the optimal dose is typically as close to the polymerization threshold as possible to maximize resolution.

The parameters were evaluated by fabricating arrays of cell traps using different combinations of p and v. Figure S1 presents an example with the corresponding printing values. The power values used were not equidistant, as the AOM controlling the laser output power exhibits a nonlinear behavior between the AOM setting and the resulting output power, so each value was measured with a power meter separately. As expected, structures printed at lower laser powers were either invisible or significantly deformed. From 34 mW onward, only minor deviations were observed, with structures appearing consistent at higher power settings. Based on this, a power slightly above 34 mW was selected for the final experiments.

Apparently, the stage velocity had a minimal impact on the visual quality of the traps. This could be due to the small size of the traps, which prevents the piezo stage from reaching its maximum speed before stopping for the next movement. Given the absence of negative effects and the benefit of shorter printing times, the highest velocity was selected for further experiments.

2. ASSESSING TRAP DIMENSIONS WITH OPTICAL TWEEZERS

Since assessing the internal structure of the printed cell traps using optical methods is challenging, we used silica beads as probe particles to determine if they remain firmly held within the traps or are easily removed. We selected silica beads with a diameter of 5 µm, closely matching the average size of the yeast cells used in the actual experiments. Cell trap structures with various size configurations were immersed in a solution of silica beads in distilled water. A tightly focused laser beam was then employed to manipulate the beads and insert them into the cell trap openings. Details of the tweezer setup are provided in reference [32] of the main manuscript. Examples of the trapping process are shown in Video 3 of the supplementary material.

By inserting beads into traps with different front-opening dimensions, we determined the minimum size required for successful bead insertion. Using different laser power settings, we attempted to displace the beads from the traps. Testing movement in all spatial directions confirmed that the beads could not escape through the rear openings or through the upper part in z-direction, indicating a solid seal of the structures.

However, no consistent correlation was observed between trap dimensions and how firmly the beads were held. Therefore, the lengths of the cell traps, which should not significantly affect cell entry into the trap, were optimized to minimize printing time.

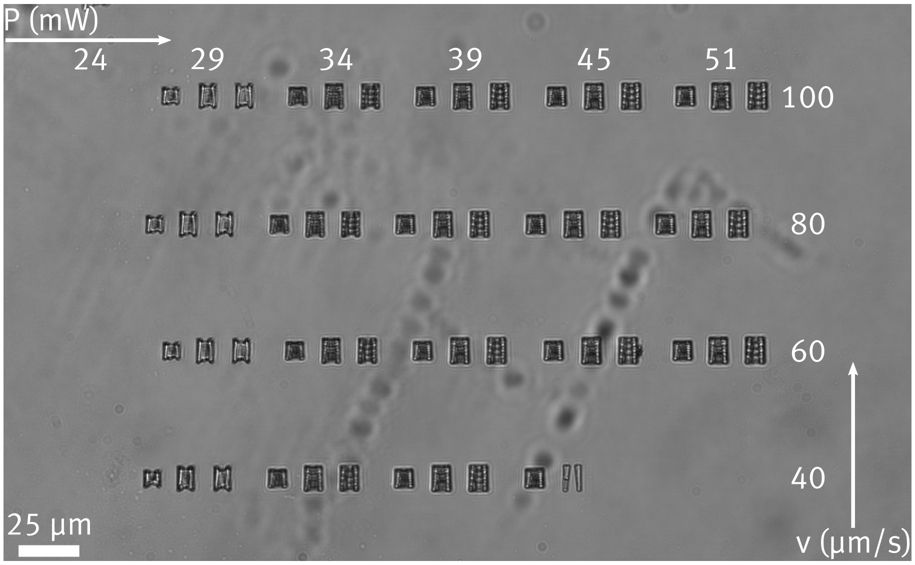

Fig. S1. Widefield microscopy image of cell traps with varying sizes, fabricated using different combinations of mean laser power p and maximum stage velocity v during the fabrication process. The printing of the array was halted before completion, so that the last structures at $v = 40\,\mu m/s$ are missing.

3. SUPPLEMENTARY VIDEOS

Movie 1 Demonstrates yeast cells trapped inside conical cell traps, exhibiting significant motion, including lateral movement, rotation, and jittering. The scale bar represents 10 μm.

Movie 2 Demonstrates yeast cells trapped inside wedge-shaped cell traps. Compared to Movie 1, the motion of the cells is noticeably suppressed. The scale bar represents 10 μm.

Movie 3 Shows three examples of the process in which silica beads are inserted into cell traps with varying configurations using optical tweezers. The red dot marks the focal spot of the trapping laser. The scale bar represents 10 μm.
